# Thioridazine Induces Cardiotoxicity via Reactive Oxygen Species-Mediated hERG Channel Deficiency and L-Type Calcium Channel Activation

**DOI:** 10.1155/2020/3690123

**Published:** 2020-01-22

**Authors:** Yan Liu, Xueqi Xu, Yuhao Zhang, Mingzhu Li, Jiamengyi Guo, Caichuan Yan, Fang Wang, Yuexin Li, Yunqi Ding, Baoxin Li, Pan Fan

**Affiliations:** ^1^Department of Pharmacology, College of Pharmacy, Harbin Medical University, Harbin, China; ^2^Department of Ophthalmology, The Second Affiliated Hospital, Harbin Medical University, Harbin, China

## Abstract

Thioridazine (THIO) is a phenothiazine derivative that is mainly used for the treatment of psychotic disorders. However, cardiac arrhythmias especially QT interval prolongation associated with the application of this compound have received serious attention after its introduction into clinical practice, and the mechanisms underlying the cardiotoxicity induced by THIO have not been well defined. The present study was aimed at exploring the long-term effects of THIO on the hERG and L-type calcium channels, both of which are relevant to the development of QT prolongation. The hERG current (*I*_hERG_) and the calcium current (*I*_Ca‐L_) were measured by patch clamp techniques. Protein levels were analyzed by Western blot, and channel-chaperone interactions were determined by coimmunoprecipitation. Reactive oxygen species (ROS) were determined by flow cytometry and laser scanning confocal microscopy. Our results demonstrated that THIO induced hERG channel deficiency but did not alter channel kinetics. THIO promoted ROS production and stimulated endoplasmic reticulum (ER) stress and the related proteins. The ROS scavenger N-acetyl cysteine (NAC) significantly attenuated hERG reduction induced by THIO and abolished the upregulation of ER stress marker proteins. Meanwhile, THIO increased the degradation of hERG channels via disrupting hERG-Hsp70 interactions. The disordered hERG proteins were degraded in proteasomes after ubiquitin modification. On the other hand, THIO increased *I*_Ca‐L_ density and intracellular Ca^2+^ ([Ca^2+^]_i_) in neonatal rat ventricular cardiomyocytes (NRVMs). The specific CaMKII inhibitor KN-93 attenuated the intracellular Ca^2+^ overload, indicating that ROS-mediated CaMKII activation promoted calcium channel activation induced by THIO. Optical mapping analysis demonstrated the slowing effects of THIO on cardiac repolarization in mouse hearts. THIO significantly prolonged APD_50_ and APD_90_ and increased the incidence of early afterdepolarizations (EADs). In human induced pluripotent stem cell-derived cardiomyocytes (hiPSC-CMs), THIO also resulted in APD prolongation. In conclusion, dysfunction of hERG channel proteins and activation of L-type calcium channels via ROS production might be the ionic mechanisms for QT prolongation induced by THIO.

## 1. Introduction

Thioridazine (THIO) is a phenothiazine derivative that has been used for the management of major psychotic disorders over the past few decades [1]. In addition, THIO also exhibits anticancer, antimicrobial, and antiviral activities, and its role in combinational drug application is being actively investigated [2–4]. However, its potential clinical applications are largely limited by its severe cardiotoxicity, including significant prolongation of the QT interval on the electrocardiogram and an increase in the propensity of developing torsades de pointes (TdP) or sudden cardiac death in patients receiving clinically effective doses of THIO [5, 6]. Identification of the molecular mechanisms underlying THIO-induced cardiotoxicity is therefore an important objective for improved treatment of clinical patients.

Assessing the effects of drugs on multiple cardiac ion channels is a major component of the Comprehensive in vitro Proarrhythmia Assay (CiPA), an ongoing guideline for evaluating drug cardiosafety [7]. The most common underlying mechanism for drug-induced QT prolongation is the inhibition of the hERG (human ether-a-go-go-related gene) potassium channel (including acute blockage and a decrease in the density of mature channels on the cell membrane), which plays a crucial role in phase 3 repolarization of cardiac action potential [8]. Previous studies have demonstrated that THIO acutely blocks the hERG channel and the binding site is at F656 of the hERG sequence [9, 10]. However, whether THIO chronically regulates the hERG expression has not been investigated. hERG channels are transcribed in the nucleus; after folding and assembly properly with the assistance of chaperones at the ER (135 kDa), hERG channels enter the Golgi for proper glycosylation (155 kDa) and are finally trafficked to the cell membrane for function [11]. The density of hERG channels on the plasma membrane is controlled by a balance between anterograde trafficking and retrograde degradation [12]. Here, we demonstrate that THIO reduces hERG membrane expression by inhibiting forward trafficking from the ER to the cell surface and subsequently promoting mature channel degradation.

In another respect, increases in cardiac calcium currents during the plateau phase of action potential can also result in QT prolongation [13]. Enhancement of L-type Ca^2+^ current (*I*_Ca‐L_) can be facilitated by calmodulin-dependent protein kinase II (CaMKII) activation in response to oxidative stress [14–16]. We found in our pilot study that THIO also increased the production of reactive oxygen species (ROS) in cultured neonatal rat ventricular myocytes (NRVMs). These findings together prompted us to hypothesize that activation of CaMKII by increasing ROS levels in cardiomyocytes might be an important factor in THIO-induced abnormal QT interval prolongation that underlies the cardiotoxicity associated with the use of this compound. The present study was set to examine this hypothesis.

## 2. Materials and Methods

### 2.1. Cell Culture

hERG-HEK293 cells (kindly provided by the Montreal Heart Institute, Canada) were cultured in Dulbecco's modified Eagle's medium (DMEM, HyClone, USA) supplemented with 10% fetal bovine serum (FBS, Bioind, Israel) and 400 *μ*g·mL^−1^ gentamycin (G418, Invitrogen, USA) with 5% CO_2_ at 37°C.

hiPSCs differentiated into cardiomyocytes as described previously [17]. Before electrophysiological analysis, hiPSC-CMs were reseeded on glass coverslips which were precoated with 500 *μ*L of a 1 : 100 diluted Matrigel solution (BD Biosciences, USA) overnight in 24-well plates. After cells resume beating, Cardiomyocyte Maintenance Medium (CellapyBio, Beijing, China) was refreshed every two days.

Neonatal rat ventricular myocytes (NRVMs) were isolated from dissected hearts of 1-2-day-old neonatal Sprague-Dawley rats (provided by the Experimental Animal Center of the Second Affiliated Hospital of Harbin Medical University, China). Cardiomyocytes were cultured as described previously [18].

### 2.2. Cellular Electrophysiology

hERG currents were measured using the whole-cell patch clamp techniques. After incubation with THIO for 24 h, hERG-HEK293 cells were trypsinized, centrifuged, and stored in Tyrode's solution at 4°C for 1 h to stabilize separated single cells. The cells were subsequently seeded in a recording chamber on a microscope (Olympus, Japan) for 10 min. Tyrode's solution contained 136 mM NaCl, 5.4 mM KCl, 5 mM HEPES, 1 mM MgCl_2_·6H_2_O, 1 mM CaCl_2_, and 10 mM glucose (pH 7.4 with NaOH). Patch pipettes contained 130 mM KCl, 1 mM MgCl_2_·6H_2_O, 10 mM HEPES, 5 mM Mg-ATP, 5 mM EGTA, and 0.1 mM GTP (pH 7.3 with KOH) and had resistances of 2-4 MΩ. The current was recorded with an Axopatch-200B amplifier (Axon Instruments Inc., Union City, CA, USA). Computer software (Clampex 9.2; Axon Instruments, USA) was used to generate voltage-clamp protocols and acquire data.

To measure steady-state inactivation properties of hERG channels, the membrane potential was inactivated at a holding potential of +40 mV; then, the resulting peak outward current at constant +20 mV was recorded after the membrane potential was recovered from -120 to +20 mV in 10 mV increments for 10 ms. We used a three-pulse protocol to test the effect on the onset of inactivation of the hERG current. The channel was depolarized at a holding potential of +40 mV; then, a prepulse to -100 mV was applied to restore the inactivated channel to an open state. Following the recovery prepulse, a series of test pulses were delivered to potentials ranging from -120 to +30 mV for 85 ms, and the outward inactivating currents were recorded. To measure the time constant of recovery from inactivation, a two-pulse protocol was used. The cells were held at a 2.48 s depolarizing pulse to +40 mV to inactivate the hERG channels and then repolarized to voltages between -120 mV and +30 mV for 90 ms to yield tail currents.

L-type calcium currents (*I*_Ca‐L_) were recorded from NRVMs with a pipette solution containing 20 mM CsCl, 110 mM CsOH, 1 mM MgCl_2_·6H_2_O, 10 mM HEPES, 5 mM Mg-ATP, 5 mM EGTA, and 110 mM aspartate (pH 7.3 with CsOH). The extracellular solution contained 136 mM Tris-Cl, 5.4 mM CsCl, 2 mM CaCl_2_, 1 mM MgCl_2_, 10 mM HEPES, and 5 mM glucose (pH 7.4 with Tris-OH). Current-voltage relationships were determined through 400 ms voltage steps ranging from -60 to +60 mV in 5 mV increments from a holding potential of -50 mV. Peak *I*_Ca‐L_ values were expressed as current density by normalizing to cell capacitance (pA/pF) and presented as mean ± SEM.

Action potentials were recorded from hiPSC-CMs with a pipette solution containing 120 mM KCl, 1 mM MgCl_2_·6H_2_O, 10 mM HEPES, 3 mM Mg-ATP, and 10 mM EGTA (pH 7.3 with KOH). The extracellular solution is Ca^2+^-containing Tyrode's solution.

Since *I*_hERG_ and *I*_Ca‐L_ both are activated during the plateau phase of cardiac action potential, it is hard, if not impossible, to completely separate these two currents to avoid mutual contaminations. Pharmacological separation of these currents could be achieved; however, adding an extra agent could confound the true effects of THIO. We therefore chose to investigate *I*_hERG_ in HEK293 cells with stable hERG overexpression and minimal contaminating ion current. By the same reason, we chose to study *I*_Ca‐L_ in NRVMs because these cells express minimal hERG channel proteins. On the other hand, to record typical cardiac action potentials, we used hiPSC-CMs that possess electrophysiological properties of human cardiomyocytes.

### 2.3. Optical Mapping

Kunming (KM) male mice (25-35 g) were used for optical mapping studies. All animal care and experimental procedures were approved by the Animal Care and Use Committee of Harbin Medical University and were in accordance with the guidelines of the Chinese Council on Animal Protection. After intragastric administration of 25 mg/kg THIO or salines for 7 days, mice were killed by cervical dislocation and the hearts were removed quickly to the oxygenated Tyrode's solution (37 ± 1°C). Tyrode's solution contained 128.2 mM NaCl, 1.8 mM CaCl_2_, 4.7 mM KCl, 1.05 mM MgCl_2_, 1.19 mM NaH_2_PO4, 20 mM NaHCO_3_, and 11.1 mM D-glucose (pH 7.36~7.39). Then, the aorta was attached to a cannula and fixed with a silk suture. The heart was perfused with Tyrode's solution. Blebbistatin (4 *μ*L, 2.5 mg/mL in DMSO, Tocris Bioscience, St. Louis, MO) was added to perfusate to prevent the cardiac contractions. Then, the voltage-sensitive dye RH237 (30 *μ*L, 2.5 mg/mL in DMSO, Invitrogen, Carlsbad, CA) was added to 1 mL Tyrode's solution and slowly injected to the perfusate. After 10 min, fluorescence images were captured with a CMOS camera (SciMedia MiCAM ULTIMA), at a resolution of 100 × 100 pixels at a rate of 1000 frames/s for 3 s. Steady-state ventricular APs were evoked by electric pacing at cycle lengths of 100, 120, 150, and 200 ms.

### 2.4. Western Blot Analysis

Cells were placed on ice and washed three times with PBS. After PBS was sucked up cleanly with a filter paper, 80 *μ*L RIPA (Beyotime, China) and 0.8 *μ*L PMSF (Beyotime, China) were added to the flask. The cells were scraped from the flask and lysed in lysis buffer. Then, the cell lysates were collected into tubes, ultrasonically oscillated 3 times, and centrifuged at 12000 rpm for 15 min to obtain protein supernatants. After adding loading buffer (Beyotime, China), the proteins (100 *μ*g per sample) were then separated by SDS-PAGE and transferred to a PVDF membrane. For the detection of oxidized CaMKII, *β*-mercaptoethanol was omitted during the denaturation process in order to avoid unspecific reduction of oxidized CaMKII. The membrane was blocked with 5% nonfat milk at room temperature for 2 h and probed with corresponding primary antibodies at 4°C overnight. Next day, the membrane was incubated with secondary antibodies in the dark for 1 h. After washing, the bands were detected with the Odyssey instrument (American Gene Corporation). Image Studio Software was used for analyzing the blots.

### 2.5. Immunoprecipitation

A total of 1.0 mg whole-cell proteins with 2.0 *μ*g appropriate primary antibody was placed on a 360° shaker at 4°C overnight. Subsequently, 30 *μ*L protein A/G PLUS agarose beads (Santa Cruz) was added to the mixture on a 360° shaker overnight. The beads were washed three times with 500 *μ*L ice-cold TBST buffer. Finally, 150 *μ*L 1× loading buffer was added to the precipitations, boiled for 8 min, and centrifuged at 1500 rpm for 5 min. The supernatants were sucked up for Western blot analysis.

### 2.6. Immunofluorescence

hERG-HEK293 cells were seeded on polylysine-coated coverslips with proper density overnight. The cells were cultured in control conditions or exposed to 3 *μ*M THIO for 24 h. Coverslips were washed 2~3 times with PBS, fixed in 4% paraformaldehyde for 30 min, permeabilized with 0.4% Triton X-100 for 1 h, and blocked with 10% goat serum (Boster, China) for 1 h. Permeabilized cells were incubated with an hERG primary antibody at 4°C overnight. On the following day, the cells were incubated with diluted Alexa Fluor 488 (Molecular Probes) in the dark at 37°C for 1 h and then DAPI (Beyotime, China) for 10 min. Finally, cells on coverslips were imaged under a fluorescence microscope.

### 2.7. Measurement of Intracellular ROS

Cells were seeded in 10 mm × 10 mm dishes and cultured with THIO for 24 h, and ROS level was detected using the Reactive Oxygen Species Assay Kit (Beyotime, China). At the end of the treatment, the cells were incubated with 10 *μ*M oxidation-sensitive fluorescent probe DCFH-DA at 37°C for 20 min. Then, the cells were washed three times with serum-free cell culture medium to fully remove DCFH-DA. The relative ROS levels were measured using a laser scanning confocal microscope (Olympus, Japan), and oxidized DCFDA fluorescence was quantified by the image analyzer ImageJ. The relative ROS levels in cells were also measured using flow cytometry at 488 nm excitation and 525 nm emission.

### 2.8. Measurement of Intracellular Calcium Concentration ([Ca^2+^]_i_)

NRVMs cultured on a 24-well plate were treated with THIO for 24 h after deprivation of serum for 12 h. Intracellular Ca^2+^ imaging of ventricular cardiomyocytes loaded with Fluo-3/AM (Invitrogen) was performed with a confocal laser scanning microscope (Olympus FV300) with 488 nm beam for excitation from an argon ion laser and 530 nm beam for emission. The sample was scanned every 3 s for about 300 s in normal Ca^2+^-containing Tyrode's solution. KCl (30 mM) was added for 60 s. The magnitude of fluorescent signals was quantified in terms of FI/F0, where F0 is the fluorescence of the basal level and FI is the value after drug administration.

### 2.9. Agents and Antibodies

A rabbit anti-hERG antibody, mouse anti-GRP78 antibody, mouse anti-PDI antibody, and mouse anti-ubiquitin antibody were purchased from Santa Cruz. Rabbit anti-IgG antibodies were purchased from Elabscience Biotechnology (Wuhan, China). A mouse anti-*β* actin antibody was purchased from ZSBG-Bio (Beijing, China). A mouse anti-Hsp70 antibody, a mouse anti-ATF6 antibody, and rabbit anti-calnexin and anti-calreticulin antibodies were purchased from Abcam. A rabbit anti-ox-CaMKII antibody was purchased from Millipore. A rabbit anti-CaMKII (pan) antibody was purchased from Cell Signaling Technology.

Thioridazine (Sigma) and N-acetyl cysteine (Biosharp, China) were dissolved in double-distilled water. Brefeldin A (Beyotime, China) was dissolved in ethanol. Drug stock solutions were diluted in DMEM before use.

### 2.10. Statistical Analysis

Data are presented as mean ± standard error of the mean (SEM). GraphPad Prism 5.0 was used for data analysis. One-way ANOVA with Bonferroni's posttest and Student's *t*-test were used to identify the differences among and between the means, respectively. *P* values < 0.05 were considered statistically significant.

## 3. Results

### 3.1. THIO Reduces the Expression of Mature hERG Protein and hERG Current in a Concentration-Dependent Manner

**Figure 1:**
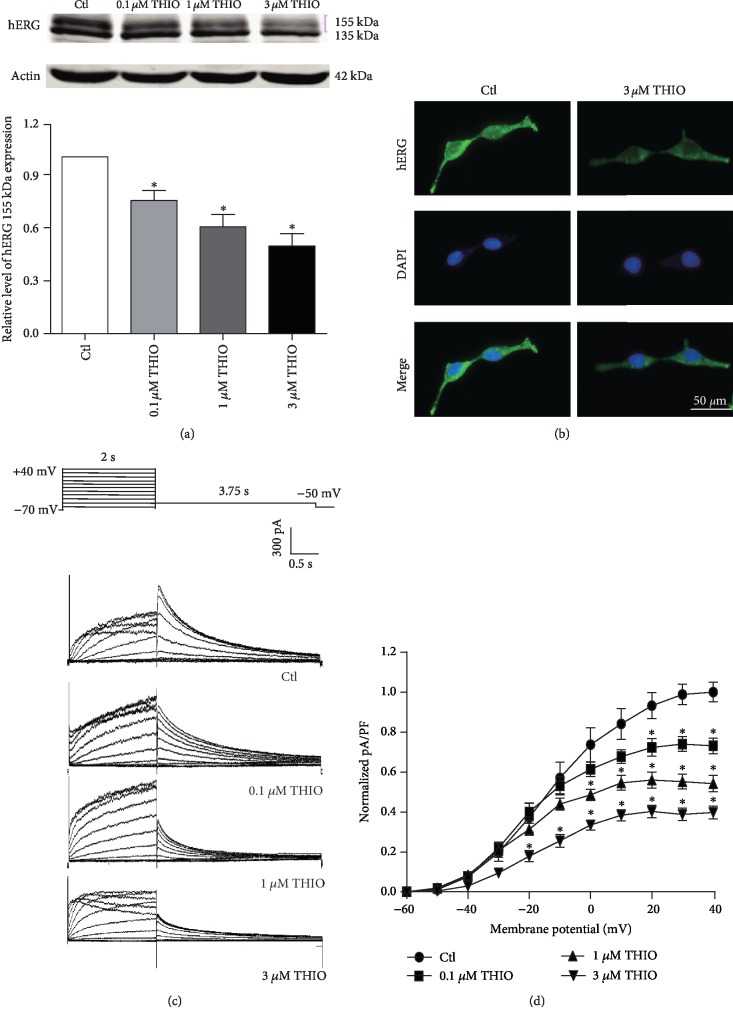
THIO treatment reduces hERG protein levels and hERG current. (a) Western blot results and statistics for hERG expression in the absence or presence of THIO (incubation for 24 h). THIO significantly reduced 155 kDa of the hERG channel in a concentration-dependent manner (0.1, 1, and 3 *μ*M) (*n* = 5). (b) Immunofluorescence showed reduced hERG protein expression by incubation with 3 *μ*M THIO. Scale bar, 50 *μ*m. (c) Protocol and examples on the hERG current under control or THIO-treated conditions. (d) *I*-*V* curve of the hERG current. THIO concentration dependently reduced the hERG current (*n* = 11). ^∗^*P* < 0.05 vs. control.

To determine the long-term effect of THIO on the hERG channels, hERG-HEK293 cells were treated with increasing concentrations of THIO (0.1, 1, and 3 *μ*M) for 24 h. hERG expression was examined by Western blotting. As shown in Figure 1(a), the protein levels of the mature 155 kDa hERG channel subunit were significantly decreased in a concentration-dependent manner: THIO reduced the band density by 24.76% ± 5.74% (0.1 *μ*M), 39.68% ± 6.98% (1 *μ*M), and 50.40% ± 7.08% (3 *μ*M). Consistent with these findings, immunofluorescence analyses (Figure 1(b)) showed that 3 *μ*M THIO significantly reduced mature hERG protein (green on the cell membrane).

To further explore whether the reduction of mature hERG protein causes a dysfunction in the hERG currents, we analyzed hERG currents recorded from hERG-HEK293 cells, which had been incubated with different concentrations of THIO for 24 h. Before recording, THIO was washed out for 2 hours in order to completely rule out its acute effect on hERG channels. In Figures 1(c) and 1(d), the hERG current was decreased by THIO in a concentration-dependent manner. In the presence of THIO, hERG current density (at +40 mV) was decreased by 26.94 ± 3.87% (0.1 *μ*M), 45.85 ± 4.11% (1 *μ*M), and 60.29 ± 3.22% (3 *μ*M) relative to the control group.

### 3.2. Long-Term Treatment with THIO Does Not Affect hERG Channel Kinetics

**Figure 2:**
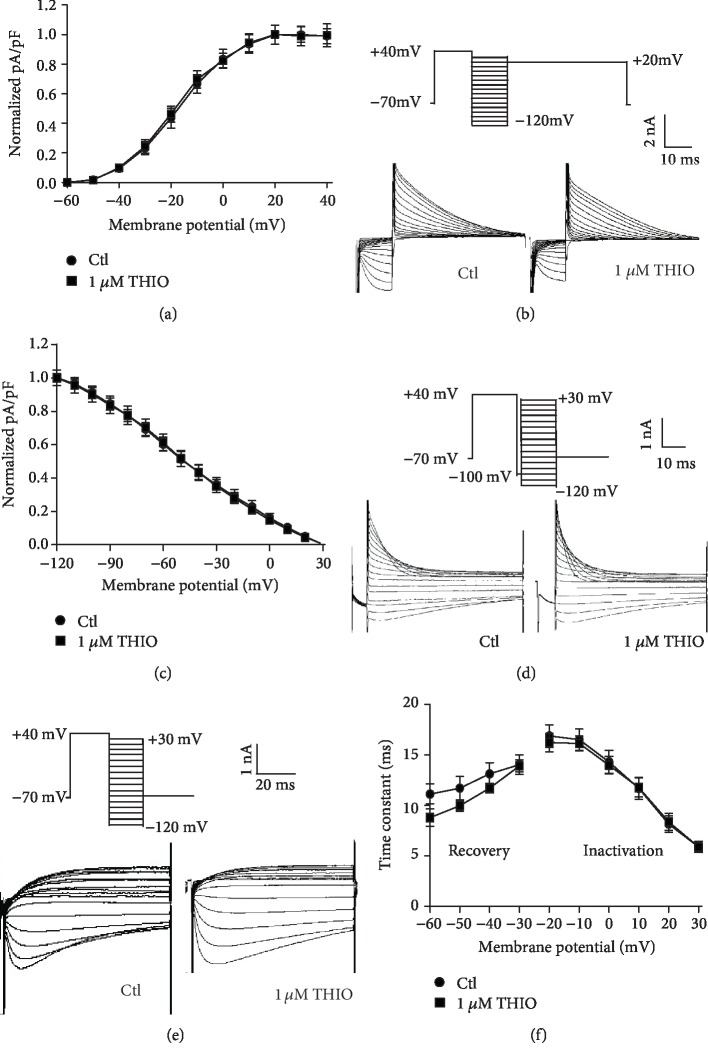
The effect of THIO on hERG channel kinetics. (a) Voltage-dependent activation curves for the control group and group following exposure to 1 *μ*M THIO for 24 h. Curves were best fits of the data to a Boltzmann function. (b) Voltage clamp protocol and representative current tracing for steady-state inactivation. (c) Normalized steady-state inactivation curves before and after exposure to 1 *μ*M THIO. (d) Voltage clamp protocol and representative current tracing for the onset of inactivation. (e) Voltage clamp protocol and representative current tracing for the recovery from inactivation. (f) The effect of 1 *μ*M THIO on the time constant for the onset of inactivation and recovery from inactivation after incubation for 24 h. *n* = 10.

hERG-HEK293 cells were used to detect the long-term effects of THIO on hERG channel kinetics. The activation curves of *I*_hERG_ were constructed by normalizing the tail currents recorded with the protocol used in Figure 1(c). The normalized data were plotted against the test pulse potentials and fitted to the Boltzmann function (Figure 2(a)). The rate of channel activation showed no significant changes after incubation with 1 *μ*M THIO for 24 h, compared to the control group. *V*_1/2_ values were −17.60 ± 1.95 mV for control and −18.87 ± 2.04 mV for THIO (*P* > 0.05).  Figure 2(b) shows the representative current traces for steady-state inactivation using a double-pulse protocol. In Figure 2(c), the inactivating outward current amplitude was normalized and plotted against the test pulse potential, giving a steady-state inactivation curve. This curve could be fitted with a Boltzmann distribution, yielding inactivation *V*_1/2_ and *k* values. THIO at 1 *μ*M did not significantly shift the inactivation curve: *V*_1/2_ was −51.46 ± 6.27 mV for the control group and −49.50 ± 6.04 mV for the 1 *μ*M THIO group. The corresponding *k* values were −42.79 ± 10.37 for control and −38.55 ± 9.50 for 1 *μ*M THIO. Additionally, we also assessed the effect of THIO on the onset of inactivation and recovery from inactivation. The effect on the onset of inactivation of the hERG current was investigated using a three-pulse protocol. With the instantaneous *I*-*V* protocol, currents for the onset of inactivation were recorded (Figure 2(d)). The time constant for the onset of inactivation was obtained by fitting a single exponential function to the decaying current traces during the third pulse of the protocol.  Figure 2(f) shows that inactivation was not changed by 1 *μ*M THIO at test potentials ranging from -20 to +30 mV.

To determine recovery from inactivation, the fully activated *I*-*V* protocol shown in Figure 2(e) was used. The time constant for recovery from inactivation was determined by fitting a single exponential function to the initial increase in tail current amplitude at potentials between -60 and -20 mV.  Figure 2(f) shows that the differences in the time constants for recovery between the control group and the cells exposed to 1 *μ*M THIO were not significant. Similarly, 3 *μ*M THIO did not significantly alter the kinetics parameters of the hERG channel ([Supplementary-material supplementary-material-1]).

These results indicate that THIO inhibited the hERG current without altering the kinetics of channel gating, suggesting that THIO-mediated hERG channel damage is dependent on a decrease in protein levels.

### 3.3. THIO Induces Endoplasmic Reticulum Stress Response in hERG-HEK293 Cells

**Figure 3:**
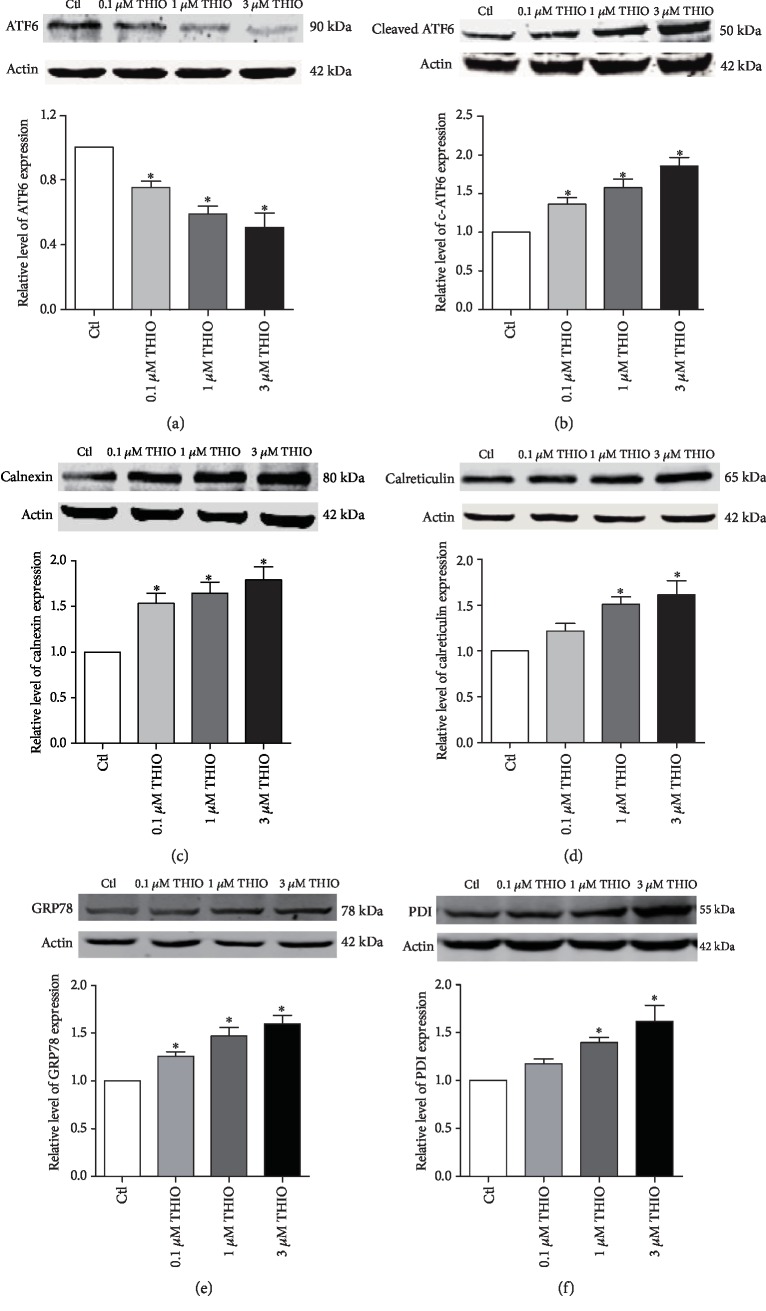
ER stress is activated by THIO. (a, b) Expression of ATF6 (~90 kDa) and cleaved ATF6 (~50 kDa) after THIO incubation for 24 h. THIO significantly improved the expression of cleaved ATF6, while ATF6 was logically reduced. ^∗^*P* < 0.05 vs. control. *n* = 4. (c–f) Representative bands and statistics of calnexin, calreticulin, GRP78, and PDI. THIO increased the expression of these four chaperones. ^∗^*P* < 0.05 vs. control. *n* = 5.

Endoplasmic reticulum (ER) stress is one of the important factors that cause protein processing disturbances. We therefore went on to determine if THIO could induce ER stress by detecting the expression of activating transcription factor 6 (ATF6), a marker protein of the ER stress [19]. As depicted in Figures 3(a) and 3(b), THIO significantly downregulated the protein level of total ATF6 (~90 kDa) and upregulated the cleaved ATF6 (~50 kDa) that could presumably activate the ER stress-responsive genes.

Once the cleaved ATF6 translocate to the nucleus, where they stimulate the transcription of UPR genes, such as glucose-regulated protein 78 (GRP78) and protein disulfide isomerase (PDI). Given that calnexin and calreticulin are the downstream targets of cleaved ATF6 and they play an important role in the ER quality control pathways [20], we decided to test whether the expression of these downstream effectors was altered by THIO treatment. As illustrated in Figures 3(c)–3(f), the expression of calnexin, calreticulin, GRP78, and PDI was significantly increased. These findings suggest that THIO can activate the ER stress.

### 3.4. THIO-Induced ER Stress Is Mediated by ROS Production

**Figure 4:**
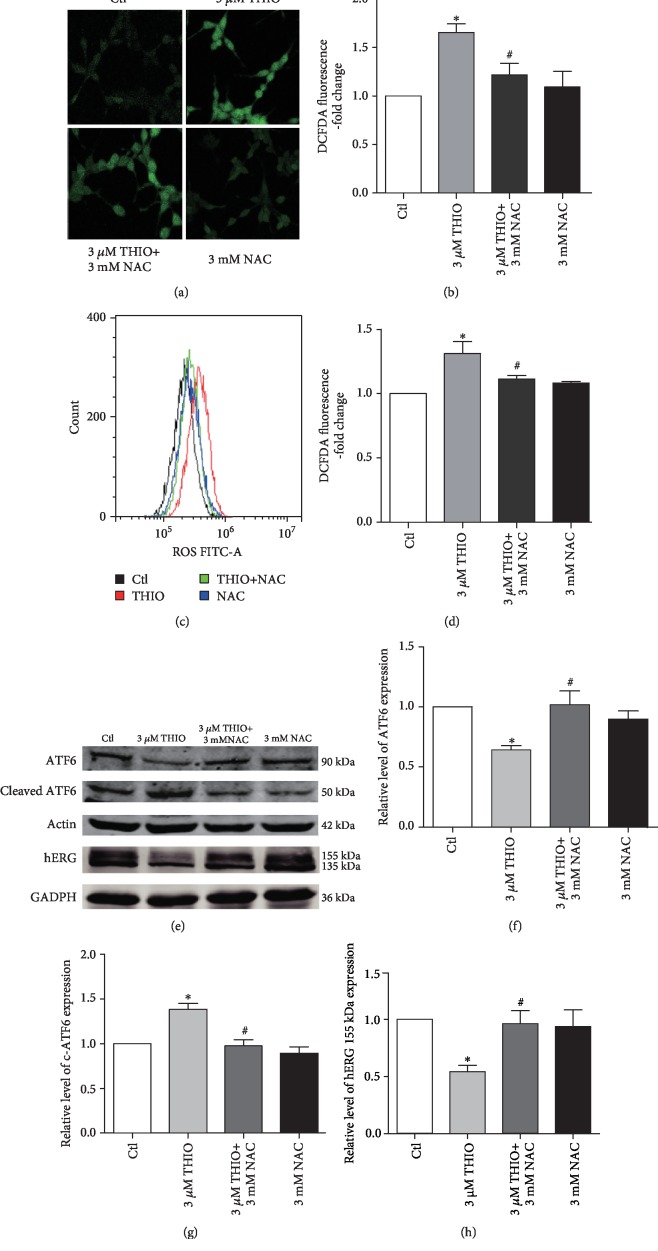
ROS generation mediates the ER stress pathway induced by THIO. (a) Macrographs of DCFDA fluorescence were detected with the confocal microscope. THIO induced a significant increase in the production of ROS, which was significantly decreased in hERG-HEK293 cells pretreated with NAC for 1 h. Original magnification, ×200. (b) Values for ROS production were quantified using ImageJ (NIH, Bethesda, MD). Values are mean ± SEM from three independent experiments and normalized to respective controls. (c, d) hERG-HEK293 cells were treated as in (a) and the mean fluorescence intensity quantified by flow cytometry. (e–f) ATF6, cleaved ATF6, and hERG expression in hERG-HEK293 cells treated with THIO (3 *μ*M) in the presence or absence of NAC (3 mM) were detected by Western blot. *n* = 5. ^∗^*P* < 0.05 vs. control and ^#^*P* < 0.05 vs. THIO.

ROS plays a critical role in many cellular processes, and it is one of the major factors in ER stress [21]. To clarify whether ROS participates in THIO-induced ER stress, we first evaluated the effect of THIO on ROS level in hERG-HEK293 cells using the DCFH-DA method. As shown in Figures 4(a)–4(d), ROS level was considerably increased in THIO-treated hERG cells compared with the control group. This increase was prevented by pretreatment with 3 mM NAC (ROS scavenger). Next, we turned our attention to the possible association between ROS generation and THIO-induced ER stress. As expected, NAC reduced the THIO-induced elevation of cleaved ATF6 and diminishment of total ATF6. Moreover, NAC reversed the downregulation of hERG expression caused by THIO treatment (Figures 4(e)–4(h)). These results suggest that the involvement of ROS in triggering ER stress by THIO decreased the level of mature hERG channel proteins.

### 3.5. THIO Disrupts Complexes of Hsp70-hERG

**Figure 5:**
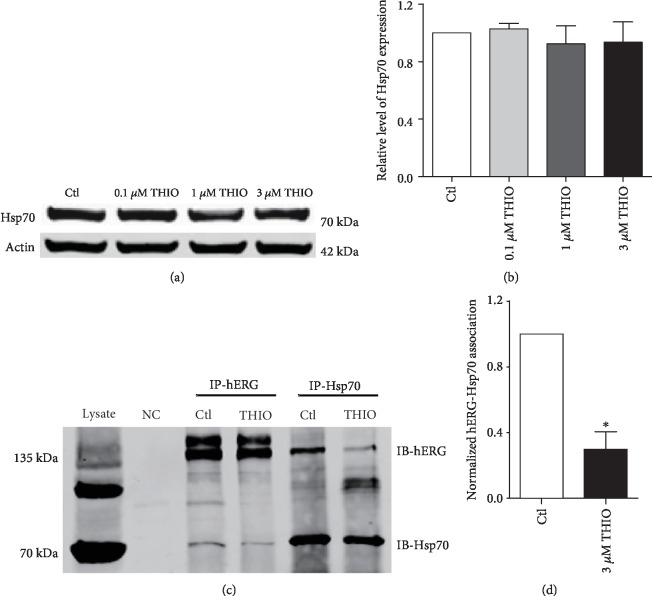
THIO reduces the interactions between hERG and Hsp70. (a, b) Western blot results for Hsp70 expression in the presence of THIO for 24 h. The expression of Hsp70 was not changed. *n* = 5. (c, d) Analysis of hERG/Hsp70 complexes formed under control conditions and in the presence of 3 *μ*M THIO. hERG/Hsp70 complexes were isolated by immunoprecipitation with anti-hERG and anti-Hsp70 antibodies. Image densities of 135 kDa hERG on Western blots were quantified. hERG image densities in immunoprecipitations with an anti-hERG antibody were used as a measure of total hERG protein. THIO reduces the formation of hERG-Hsp70 complexes. ^∗^*P* < 0.05 vs. Hsp70 control. *n* = 3.

Failure of hERG proteins to be exported out of the ER can directly trigger degradation. Hsp70 is the predominant molecular chaperone assisting in hERG protein folding at the ER [22], but once Hsp70 is dissociated from hERG, it can be recognized by certain E3 ubiquitin ligases, accelerating endoplasmic reticulum-associated protein degradation (ERAD) of hERG channels [23]. Western blot analysis shows that the expression levels of Hsp70 remained unaltered after THIO incubation (Figures 5(a) and 5(b)). We therefore turned to exploring whether THIO prevents maturation of hERG channels via inhibiting chaperone-hERG interactions; to this end, we performed coimmunoprecipitation experiments to determine the association of hERG protein with Hsp70. The relative band density of 135 kDa hERG signal identified by anti-Hsp70 antibodies over that identified by an anti-hERG antibody was determined to indicate the changes of interactions between hERG and chaperones. As illustrated in Figures 5(c) and 5(d), hERG/Hsp70 complexes were decreased in the presence of 3 *μ*M THIO. These results indicate that THIO disturbed the folding process of the hERG channel by reducing the interaction between hERG and Hsp70, eventually leading to the degradation of disordered hERG proteins.

### 3.6. THIO Induces Ubiquitination and Degradation of hERG

**Figure 6:**
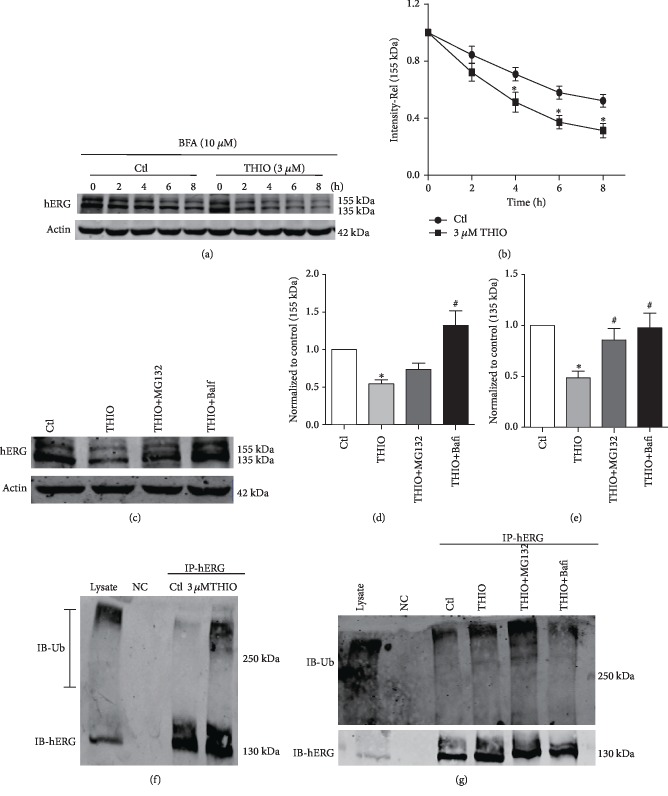
THIO accelerates the degradation of the hERG channel on the cell surface. (a) Time-dependent effects of incubating hERG-HEK293 cells with BFA (10 *μ*M) in 0 or 3 *μ*M THIO on hERG protein expression level. (b) Image densities of 155 kDa hERG bands were normalized to their values measured at *t* = 0. *n* = 5. (c–e) Impaired hERG proteins are degraded in both the lysosome and proteasome. hERG expression was detected after hERG-HEK293 cells were incubated with THIO or the proteasome/lysosome inhibitor for 24 h. Bafilomycin significantly restored the reduced 155 kDa hERG. MG132 and bafilomycin restored the reduced 135 kDa hERG. *n* = 5. (f) Immunoprecipitation results of hERG ubiquitination. Whole-cell lysates were immunoprecipitated with an anti-hERG antibody and immunoblotted using either an anti-ubiquitin or anti-hERG antibody. 3 *μ*M THIO significantly increased the ubiquitination of hERG protein. *n* = 3. (g) Immunoprecipitation results of hERG protein after treatment with the lysosome inhibitor or proteasome inhibitor for 24 h. *n* = 3. ^∗^*P* < 0.05 vs. control and ^#^*P* < 0.05 vs. THIO.

To further explore the mechanisms by which THIO affects hERG channel degradation, we conducted the following procedures. First, we assessed the effects of THIO on the degradation of mature hERG channels. Brefeldin A (BFA, 10 *μ*M) was used to block protein trafficking from the ER to the Golgi. hERG-HEK293 cells were pretreated with BFA for 1 h and then cultured in the absence or presence of THIO in the continued presence of BFA. As depicted in Figures 6(a) and 6(b), THIO accelerated the degradation rate of hERG compared with the control group without THIO treatment. After 8 h incubation with BFA, the band intensity of 155 kDa hERG was decreased by 68.7 ± 5.0% in THIO-treated cells and by 47.9 ± 4.4% in non-THIO-treated control cells.

The proteasome and lysosome are two major organelles in which proteins are eventually degraded. To elucidate the paths for the degradation of hERG proteins, the effects of the proteasome inhibitor MG132 (3 *μ*M) and the lysosome inhibitor bafilomycin (10 nM) were assessed.  Figure 6(c) demonstrates that only bafilomycin caused a significant restoration of the decreased mature hERG (155 kDa), indicating that mature hERG degradation was primarily executed by lysosomes. On the other hand, either MG132 or bafilomycin significantly restores the levels of immature hERG (135 kDa), suggesting the involvement of both proteasomes and lysosomes in the degradation of 135 kDa hERG.

Ubiquitination is an important common factor in targeting misfolded proteins to proteasomes or lysosomes [24]. Then, we determined whether the THIO-induced enhancement of hERG degradation was mediated by ubiquitination. After immunoprecipitation with an anti-hERG antibody, immunoprecipitates were blotted with either an anti-ubiquitin or anti-hERG antibody.  Figure 6(f) shows that 3 *μ*M THIO increased polyubiquitination of hERG proteins.

Furthermore, coincubation with MG132 significantly enhanced the THIO-induced increase in ubiquitylated hERG proteins, but there was a not significant increase in the ubiquitylated hERG proteins if the cells were pretreated with both THIO and bafilomycin (Figure 6(g)). These results suggest that the impaired hERG proteins were degraded in the lysosome and proteasomes, and polyubiquitin appeared to guide hERG proteins to proteasomes for degradation.

### 3.7. THIO Increases *I*_Ca‐L_ via ROS-Mediated CaMKII Activation

**Figure 7:**
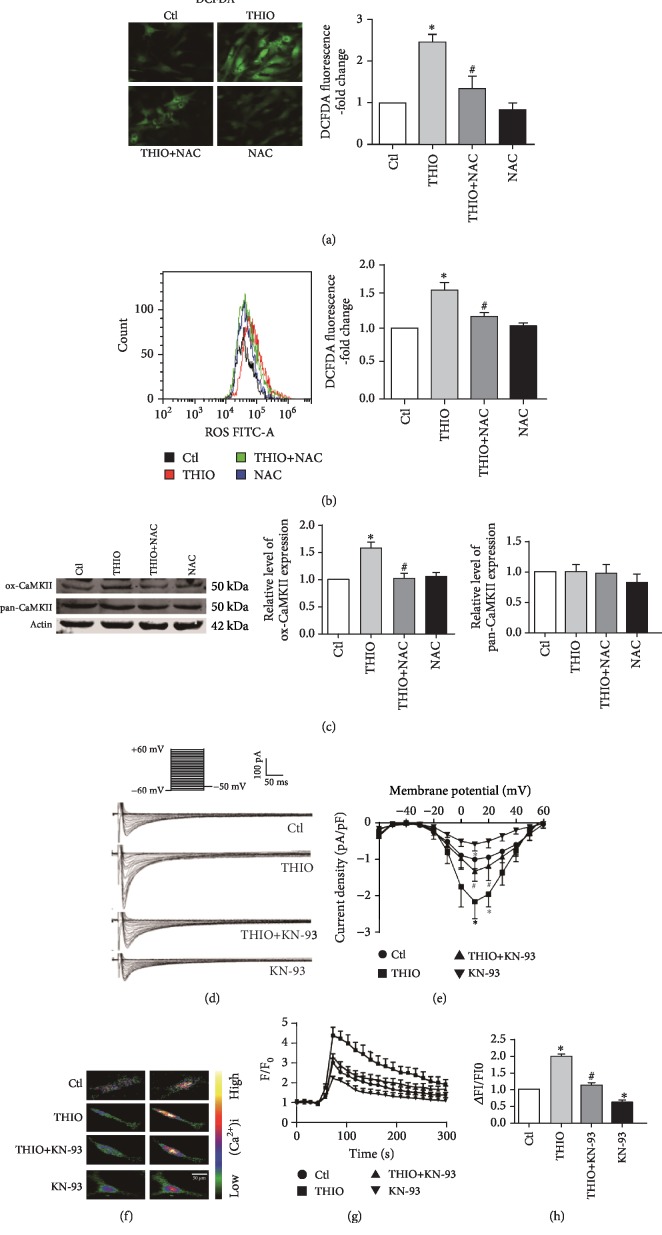
Effects of THIO (1 *μ*M, 24 h) on currents of voltage-gated L-type Ca^2+^ channels. (a) ROS accumulation as measured by DCFDA oxidation in Figure 5(a). Values are mean ± SEM from three independent experiments and normalized to control. THIO induced a significant increase in intracellular ROS, which was significantly decreased in NRVMs pretreated with NAC for 1 h. Original magnification, ×200. (b) NRVMs were treated as in Figure 5(a) and the mean fluorescence intensity was quantified by flow cytometry. (c) Representative Western blots of ox-CaMKII (top), pan-CaMKII (middle), and actin (bottom). Oxidized and total protein levels are presented relative to actin. Data are presented as the means ± SEM for each group (*n* = 5). (d) Voltage protocol and representative *I*_Ca‐L_ current. (e) *I*-*V* relationships of *I*_Ca‐L_ current (*n* = 7-9). (f) Representative confocal images of [Ca^2+^]_i_ changes before and after 30 mM KCl exposure for control (left column) and 1 *μ*M THIO (right column). (g) Mean values of the FI/F0 ratio during the 300 s scanning in the presence of 30 mM KCl. (h) The average ratio of ΔFI/FI0 in each group after 30 mM KCl stimulation. *n* = 7. Drug treatment occurred 24 h prior to analysis. ^∗^*P* < 0.05 vs. control and ^#^*P* < 0.05 vs. THIO.

While alterations of hERG function, trafficking, and/or metabolism contribute to prolongation of the cardiac action potential, changes of *I*_Ca‐L_ also play a critical role in determining action potential duration (APD) with increased *I*_Ca‐L_ being deemed to lengthen APD. We observed that the ROS level in NRVMs was substantially increased after incubation with 1 *μ*M THIO for 24 h (Figures 7(a) and 7(b)). THIO also increased the expression of ox-CaMKII, while the total CaMKII expression was unchanged. To determine whether ROS mediated the activation of CaMKII, the effect of NAC on the responses to ox-CaMKII was tested. Incubation with 3 mM NAC significantly attenuated the increase in ox-CaMKII induced by THIO (Figure 7(c)). Next, we investigated the possible involvement of CaMKII activation in the regulation of *I*_Ca‐L_ by THIO, by incubating NRVMs with 10 *μ*M KN-93, a specific CaMKII inhibitor. Our results showed that the amplitude of *I*_Ca‐L_ was significantly increased in THIO-treated cardiomyocytes at +10 mV compared with the control group. Importantly, a THIO-induced increase in *I*_Ca‐L_ density was attenuated by KN-93 (Figures 7(d) and 7(e)). It is known that *I*_Ca‐L_ plays an important role in the maintenance of [Ca^2+^]_i_, and an abnormal increase in [Ca^2+^]_i_ can lead to severe cardiac arrhythmias [25]. Therefore, we measured [Ca^2+^]_i_ using a confocal laser scanning microscope. As shown in Figures 7(f)–7(h), [Ca^2+^]_i_ in the THIO-treated group was significantly higher than that in the control group, and KN-93 significantly inhibited THIO-induced intracellular Ca^2+^ overload.

### 3.8. THIO Prolongs Action Potential Duration in Mouse Hearts and hiPSC-CMs

**Figure 8:**
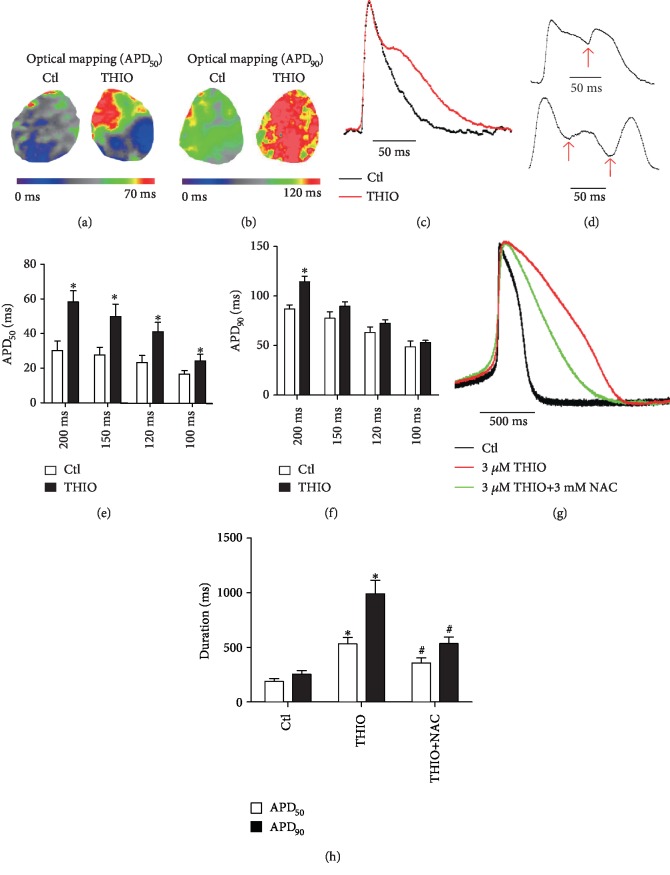
THIO induces prolongation of APD in mouse hearts and hiPS-CMs. (a, b) Action potential duration at 50% repolarization (APD_50_) and 90% repolarization (APD_90_) maps is shown from a heart under control conditions (left) and after THIO application (right). (c) Representative optical action potential traces under control (black line) and THIO (red line) conditions. (d) Representative examples of EADs in mouse hearts after THIO application. (e) Statistics of APD_50_. (f) Statistics of APD_90_. *n* = 5. (g) Representative current clamp recordings of action potentials under control conditions (black line) and 3 *μ*M THIO (red line) and 3 *μ*M THIO+3 mM NAC (green lines) treatment for 24 h in hiPS-CMs. (h) Statistics of APD_50_ and APD_90_. *n* = 5-8. ^∗^*P* < 0.05 vs. control and ^#^*P* < 0.05 vs. THIO.

Given the facts that THIO decreased *I*_hERG_ and increased *I*_Ca‐L_, it is likely that it could cause remarkable lengthening of cardiac APD. To verify this notion, we carried out optical mapping analysis of mouse hearts. The hearts were paced at cycle lengths of 100, 120, 150, and 200 ms, and optical AP traces were recorded (Figure 8(c)). The data clearly exhibit that THIO significantly prolonged APD at 50% full repolarization (APD_50_) and 90% full repolarization (APD_90_) compared to the control group. Specifically, APD_50_ was prolonged from 30.5 ± 5.55 for control to 58.6 ± 6.46 ms by THIO and APD_90_ from 86.9 ± 4.05 ms to 114.5 ± 5.50 ms at a cycle length of 200 ms.

Since THIO could produce dual effects with both APD prolongation and *I*_Ca‐L_ enhancement, it is anticipated that this compound has the potential to induce EADs and triggered activities. This notion was indeed supported by the frequent appearance of EADs in mouse hearts treated with THIO (Figure 8(d)).

Finally, we validated the effects of THIO on action potentials in hiPSC-CMs (Figures 8(g) and 8(h)). As expected, application of 3 *μ*M THIO significantly prolonged APD_50_ and APD_90_ in hiPSC-CMs. Specifically, APD_50_ was prolonged from 188.5 ± 26.35 ms to 531.6 ± 46.68 ms and APD_90_ from 254.8 ± 33.68 ms to 990.2 ± 97.36 ms. The prolonged APD_50_ and APD_90_ were shortened to 356.2 ± 44.83 ms and 533.8 ± 55.3 ms, respectively, after 3 mM NAC treatment.

## 4. Discussion

Recently, the use of antipsychotic drugs has been extended to nonschizophrenia patients, and the cases of sudden cardiac death due to lethal ventricular arrhythmias have thus been increasing. In order to prevent the occurrence of cardiotoxicity, the risk evaluations of antipsychotic treatment have become more widespread [26, 27]. In the present study, we analyzed the relevant ionic mechanisms underlying the QT prolongation induced by THIO. The long-term effects of THIO on ion currents at concentrations within the therapeutic range (i.e., 0.1–3 *μ*M) include facilitation of *I*_Ca‐L_ and inhibition of *I*_hERG_, both of which together should yield a strong force to maintain the cardiac membrane at the depolarized potentials manifested by pronounced lengthening of the plateau duration of action potential. This represents the first report to uncover the ionic mechanisms for THIO-induced QT prolongation. In addition to direct block (block of function) [10], THIO also indirectly inhibited the intracellular processes of the hERG channels (reduce channel density) after incubation for 24 h. Consistent with data obtained from the cell line, THIO treatment also decreased the expression level of rERG in neonatal rat cardiomyocytes ([Supplementary-material supplementary-material-1]). Our data further unraveled that the decrease in the hERG current is primarily a direct consequence of impairment of hERG protein trafficking and exaggeration of hERG protein degradation. The lack of effects of THIO on hERG channel kinetics suggests that the long-term effects of THIO on the hERG channel are mainly achieved by reducing the functional availability or shortening the half-life of mature channels rather than by altering the gating properties. The dual effects of THIO on *I*_hERG_ and *I*_Ca‐L_ provide a strong basis for creating deleterious effects on cardiac electrophysiology or cardiotoxicity.

It is well known that the endoplasmic reticulum has its own strict quality control system (ERQC) to supervise the productive folding process of the hERG protein [28]. When hERG channel trafficking is defective, ER stress response is often activated. Once ER stress occurs, unfolded or misfolded proteins accumulate in the ER and subsequently trigger unfolded stress response (UPR) to assist protein to complete further folding, which is possibly accompanied with the process of endoplasmic reticulum-associated protein degradation (ERAD) [29]. Three major signaling pathways characterize the UPR: IRE1, PERK, and ATF6 [30]. Among the three pathways, ATF6 target genes may be most concerned about the maintenance of ER homeostasis. During ER stress conditions, ATF6 is transported from the ER toward the Golgi where it is cleaved by site-1 and site-2 proteases (S1P and S2P), and then, the cleaved ATF6 translocates into the nucleus where it promotes transcription of UPR genes, such as chaperones BiP/GRP78 and transcription factors CHOP/XBP1, as well as other proteins such as calnexin and calreticulin to assist in hERG channel folding [31]. In our study, we found that THIO decreased inactive ATF6 and increased active-cleaved ATF6 (Figures 3(a) and 3(b)). In addition, calnexin, calreticulin, GRP78, and PDI were both upregulated (Figures 3(c)–3(f)). These results indicate that ER stress is activated by THIO-induced trafficking disorder of hERG channel proteins.

The production of ROS is an essential part of the ER stress response. ROS production can act as an upstream factor to activate ER stress, leading to cell apoptosis. In our experiments, hERG-HEK293 cells treated with THIO show increased ROS levels. Inhibition of ROS generation by NAC pretreatment decreased the expression levels of ER stress markers (Figure 4(e)), suggesting that THIO-induced ER stress was partly dependent on ROS production. Meanwhile, we also found that the decrease in hERG membrane protein caused by THIO was partially reversed by NAC, indicating that ROS acts as an upstream signaling molecule in THIO-induced ER stress activation and hERG protein reduction.

Although activation of UPR under ER stress is a protective behavior for cells, if stress persists too long, ER homeostasis cannot be restored and hERG protein will eventually undergo the degradation process [32]. The core cytosolic chaperones that assist in the protein folding by ERQC are the heat shock protein families including Hsp70. Li et al. reported that coexpression of Hsp70 increased the expression of hERG protein with a concomitant decrease in the ubiquitinated form of the protein [33]. Our coimmunoprecipitation results show that THIO aggravated the ubiquitination of the hERG protein (Figure 6(f)), and the interactions between hERG and Hsp70 were decreased (Figure 5(c)). These data provide evidence that THIO promotes hERG misfolding and degradation by inducing the dissociation of Hsp70 from hERG. Once ubiquitinated, ion channels are degraded via either proteasomal or lysosomal pathways [34]. Our subsequent test showed that bafilomycin (lysosome inhibitor) recovered 155 kDa hERG. Importantly, we found that THIO increased surface hERG degradation (Figure 6(a)), which indicates that THIO may increase lysosome-mediated mature hERG degradation. MG132 (proteasome inhibitor) and bafilomycin (lysosome inhibitor) increased 135 kDa hERG, which suggests that immature hERG undergoes both proteasome- and lysosome-mediated degradation (Figure 6(c)). Furthermore, THIO treatment in the presence of MG132 increased the steady-state level of ubiquitylated hERG protein, suggesting that THIO stimulates the degradation of the misprocessed hERG protein by the ubiquitination-proteasome pathway (Figure 6(g)).

In addition to *I*_hERG_, *I*_Ca‐L_ is another key ion current in the regulation of cardiac APD in ventricular cardiomyocytes [35]. CaMKII is a key Ca^2+^-sensing protein calmodulin (Ca^2+^/CaM) effector, which is a kind of ubiquitously expressed serine-threonine protein kinase. A growing body of evidence has demonstrated that CaMKII activation, a consequence of Met281/282 oxidation by ROS, can directly modify L-type calcium channels [36–38]. In the present study, there was a significant elevation of ROS production in NRVMs incubated with THIO. ox-CaMKII was elevated in NRVMs exposed to THIO. However, the level of ox-CaMKII was decreased after pretreatment with NAC, indicating that CaMKII functions as a downstream signal molecule of THIO (Figure 7(c)). The present study also shows that inhibition of CaMKII attenuated THIO-induced increases in *I*_Ca‐L_ and [Ca^2+^]_i_, further supporting the role of CaMKII in the enhancement of *I*_Ca‐L_ by THIO. These findings confirmed that ROS-activated CaMKII is another contributor to THIO-induced cardiotoxicity. ROS has been shown to directly induce sarcoplasmic reticulum (SR) Ca release via thiol oxidation of ryanodine receptor 2 (RyR2) [39]. In the present study, we found that the enhancement of *I*_Ca‐L_ by THIO is associated with the activation of CaMKII, although direct redox modification of L-type calcium channel subunits may also contribute. Furthermore, as shown in Figures 8(c) and 8(g), THIO prolongs both APD_50_ and APD_90_ in mouse hearts and hiPSC-CMs. Excessive lengthening of APD provides the substrate for early afterdepolarizations (EADs), and on top of that, an increase in *I*_Ca‐L_ could likely induce triggered activities leading to TdP [40, 41].

## 5. Conclusion

ROS production is a major factor accounting for THIO-induced ion channel dysfunction. ROS-induced ER stress leads to transport barriers of hERG channels; ROS-activated ox-CaMKII ultimately activates L-type calcium channels. The results of our study suggested that *I*_hERG_ downregulation and *I*_Ca‐L_ upregulation might be the primary ionic mechanisms underlying THIO-induced LQTs. Our findings also suggest that coapplication of antioxidant and THIO might be beneficial to patients with major psychotic disorders with minimal cardiotoxicity or enhanced cardiosafety.

## Data Availability

The data used to support the findings of this study are included within the article.

## Supplementary Materials

Supplementary Materials Figure S1: effect of THIO treatment on the expression of rERG in neonatal rat ventricular cardiomyocytes. Figure S2: effect of 3 *μ*M THIO on hERG channel kinetics.
