# Supplementary material for: Thioridazine Induces Cardiotoxicity via Reactive Oxygen Species-Mediated hERG Channel Deficiency and L-Type Calcium Channel Activation
Source: Oxid Med Cell Longev. 2020 Jan 22;2020:3690123. doi: 10.1155/2020/3690123 (PMC6998749; doi:10.1155/2020/3690123)
Supplement: Supplementary Materials — Figure S1: effect of THIO treatment on the expression of rERG in neonatal rat ventricular cardiomyocytes. Figure S2: effect of 3 μM THIO on hERG channel kinetics. [file 3690123.f1.pdf]

Supplementary Materials

Thioridazine induces cardiotoxicity via reactive oxygen species mediated hERG channel deficiency and L-type calcium channel activation

Yan Liu, [1] Xueqi Xu, [1] Yuhao Zhang, [1] Mingzhu Li, [1] Jiamengyi Guo, [1] Caichuan Yan, [1] Fang Wang, [1] Yuexin Li, [1] Yunqi Ding, [1] Pan Fan, [2] and Baoxin Li [1]

[1]Department of Pharmacology, College of Pharmacy, Harbin Medical University, Harbin, China

[2] The Second Affiliated Hospital, Harbin Medical University, Harbin, China

The first corresponding author: Baoxin Li

Department of Pharmacology, College of Pharmacy, Harbin Medical University,

No.157 Baojian Road, Harbin, Heilongjiang Province, 150081 (P. R. China)

(Tel): +86 451 86671354, (Fax) : +86 451 86667511

Email: libx64@hotmail.com

The second corresponding author: Pan Fan

The Second Affiliated Hospital, Harbin Medical University, No.148 Bao Jian Road, Nan Gang District, Harbin, Heilongjiang Province, 150081 (P.R. China)

Email: di20071214@163.com

Figure S1.

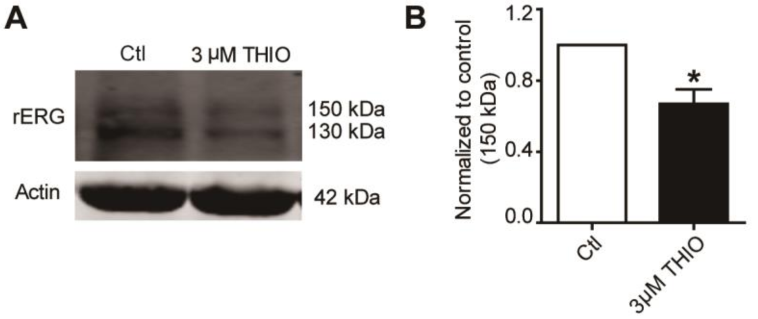

Fig. S1. Western blot results show the expression of rERG after incubation with THIO for 24 h. *P < 0.05 vs control, *n* = 4.

Figure S2.

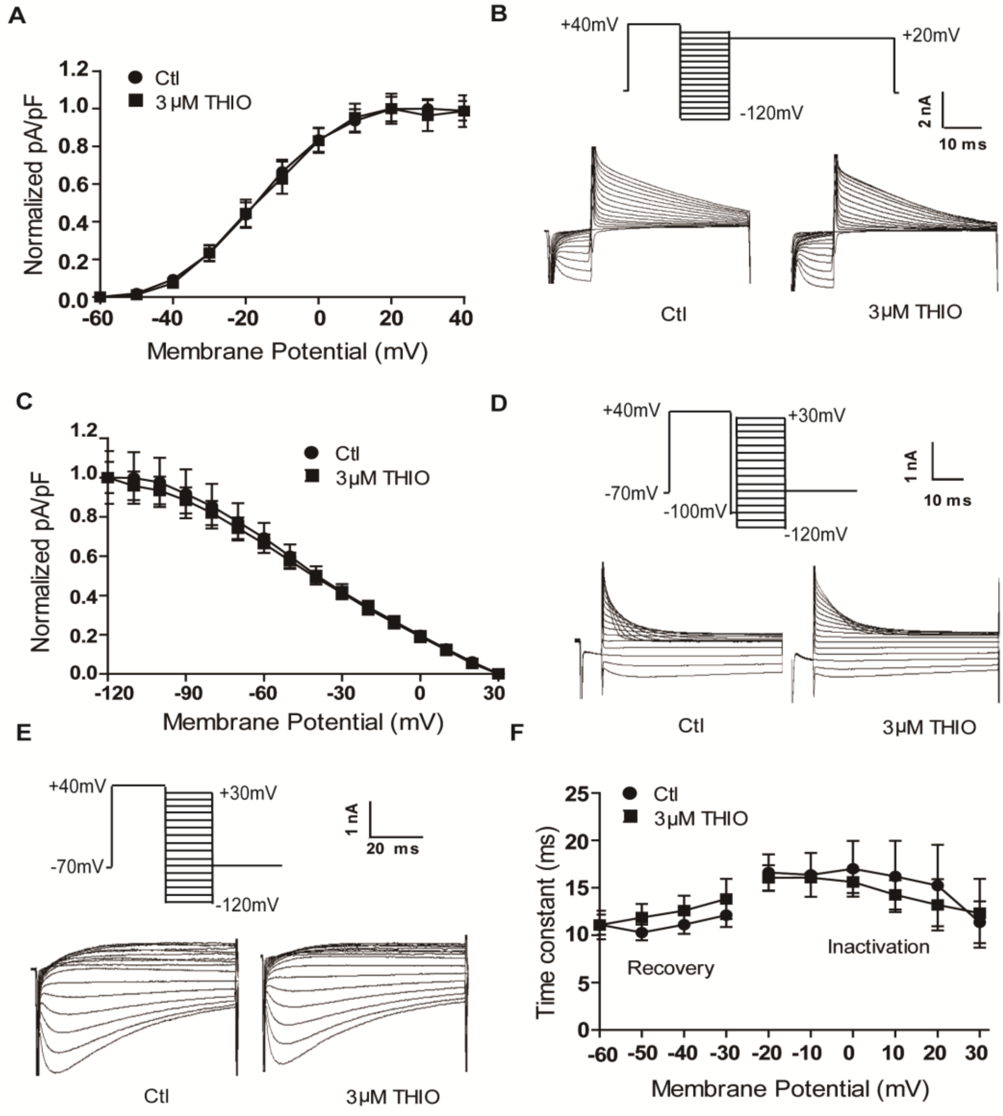

Fig. S2. **The effect of 3 μM THIO on hERG channel kinetics.** (A) Voltage-dependent activation curves for the control group and following exposure to 3 μM THIO for 24 h. Curves were best fits of the data to a Boltzmann function. (B) Voltage clamp protocol and representative current tracing for steady-state inactivation. (C) Normalized steady-state inactivation curves before and after exposure to 3 μM THIO. (D) Voltage clamp protocol and representative current tracing for the onset of inactivation. (E) Voltage clamp protocol and representative current tracing for the recovery from inactivation. (F) The effect of 3 μM THIO on the time constant for the onset of inactivation and recovery from inactivation after incubation for 24 h. n = 6.
